# Tertiary lymphoid structures in pulmonary metastases of microsatellite stable colorectal cancer

**DOI:** 10.1007/s00428-023-03577-8

**Published:** 2023-06-20

**Authors:** Topias Karjula, Anne Niskakangas, Olli Mustonen, Iiris Puro, Hanna Elomaa, Maarit Ahtiainen, Teijo Kuopio, Jukka-Pekka Mecklin, Toni T. Seppälä, Erkki-Ville Wirta, Eero Sihvo, Fredrik Yannopoulos, Olli Helminen, Juha P. Väyrynen

**Affiliations:** 1 Translational Medicine Research Unit, Medical Research Center Oulu, Oulu University Hospital and University of Oulu, Aapistie 5a, 90220 Oulu, Finland; 2 Department of Biological and Environmental Science, University of Jyväskylä, 40014 Jyväskylä, Finland; 3 Department of Education and Research, Central Finland Health Care District, 40620 Jyväskylä, Finland; 4 Department of Pathology, Central Finland Health Care District, 40620 Jyväskylä, Finland; 5 Faculty of Sport and Health Sciences, University of Jyväskylä, 40014 Jyväskylä, Finland; 6 Faculty of Medicine and Health Technology, Tampere University and TAYS Cancer Center, Tampere University Hospital, 33520 Tampere, Finland; 7 Department of Gastrointestinal Surgery, Helsinki University Central Hospital, University of Helsinki, 00290 Helsinki, Finland; 8 Applied Tumor Genomics, Research Program Unit, University of Helsinki, 00290 Helsinki, Finland; 9 Department of Gastroenterology and Alimentary Tract Surgery, Tampere University Hospital, 33520 Tampere, Finland; 10 Central Hospital of Central Finland, 40014 Jyväskylä, Finland; 11 Department of Cardiothoracic Surgery, Oulu University Hospital, Oulu, Finland

**Keywords:** Tertiary lymphoid structures, Pulmonary metastasis, Microsatellite stable colorectal cancer

## Abstract

Tertiary lymphoid structures (TLSs) are ectopic lymphoid aggregates located at sites of chronic inflammation and recognized as prognosticators in several cancers. We aimed to analyse the prognostic effect of TLSs in colorectal cancer (CRC) pulmonary metastases and primary tumours, with a comparison to the CD3^+^ and CD8^+^ cell density-based immune cell score (ICS). For TLS density and TLS maximum diameter analysis, 67 pulmonary metastases and 63 primary tumours were stained with haematoxylin and eosin. For ICS scoring and analysis, CD3 and CD8 immunohistochemistry was performed. Excellent interobserver agreement was achieved in all TLS measurements. Of all patients, 36 patients had low TLS density (< 0.222 follicles/mm) and 31 patients had high TLS density (≥ 0.222 follicles/mm) in the first resected pulmonary metastases. TLS density (adjusted HR 0.91, 0.48–1.73) or maximum diameter (adjusted HR 0.78, 0.40–1.51) did not have prognostic value in pulmonary metastases. In primary tumours, higher TLS density (adjusted HR 0.39, 0.18–0.87) and maximum diameter (adjusted HR 0.28, 0.11–0.73) were associated with lower mortality. In the pulmonary metastases, ICS had superior prognostic value to TLSs; however, TLSs and ICS were significantly associated. In conclusion, TLSs in CRC pulmonary metastases had no prognostic value but correlated with the ICS. TLSs in primary tumours associated with favourable prognosis.

**Supplementary Information:**

The online version contains supplementary material available at 10.1007/s00428-023-03577-8.

## Introduction

Lymphocyte populations in the tumour microenvironment have complex anti- and pro-tumour interactions with the cancer cells, influencing cancer progression and survival [1]. The adaptive immune response is classically thought to be activated in secondary lymphoid organs such as the lymph nodes and the spleen. However, there is accumulating evidence that an adaptive immune response can be initiated ectopically outside the secondary lymphoid organs via lymphoid neogenesis [2]. In sites of chronic inflammation such as cancer, a spectrum of lymphoid cell aggregates can be found, varying from small lymphoid cell clusters to highly organized structures with germinal centres exhibiting lymph node-like characteristics [2, 3]. These tertiary lymphoid structures (TLSs) have been reported to have prognostic value in several cancer types [4–6].

In colorectal cancer (CRC)—one of the leading causes of cancer mortality globally [7]—peritumoural cancer-associated lymphoid aggregates were named Crohn’s-like lymphoid reaction (CLR) by Graham and Appelman due to their resemblance to the lymphocytic reaction of Crohn’s disease [8]. CLR can be assessed using haematoxylin and eosin (H&E) stained sections and the evaluation can be performed as part of standard routine diagnostics. Higher CLR has been found as a prognosticator for lower risk of regional lymph node metastasis and disease recurrence, as well as longer cancer-specific and overall survival [5, 8–10].

Of all CRC patients, around 5–10% have synchronous pulmonary metastases and around 5% have disease recurrence with pulmonary metastases within 5 years after treatment of the primary tumour [11, 12]. Despite advances in cancer therapy, metastatic CRC remains a therapeutic challenge: patients with stage IV CRC at the time of diagnosis have a 5-year survival of only 14% [13]. There are few studies on TLSs in CRC metastases that demonstrate prognostic significance in pulmonary metastases [14] and liver metastases [15]; however, the studies are based on immunohistochemical analysis of individual cell types. Immunohistochemistry-based studies on tumour-infiltrating lymphocyte densities have revealed CD3^+^ and CD8^+^ T-cell density-based immune cell score (ICS) having significant prognostic value not only in the primary CRC tumour [16] but also in the liver and pulmonary metastases [17, 18]. TLSs evaluated from HE-stained sections would have practical diagnostic advantages. A need for additional classification systems is recognized as the survival within TNM stages varies significantly [19].

This study aimed to analyse the prognostic value of TLSs in resected pulmonary metastases of CRC and corresponding primary tumours, with a comparison to the CD3^+^ and CD8^+^ T-cell density-based ICS.

## Material and methods

### Study design

All patients with histologically confirmed pulmonary metastases from CRC operated in Oulu University Hospital and Central Finland Central Hospital during 2000–2020 were included in the study. This was a population-based retrospective study. The study hospitals are the only hospitals offering thoracic surgery in their hospital districts. A total of 106 pulmonary metastasectomies for CRC were performed on 74 patients during the study period in the study hospitals. Patients were considered for pulmonary metastasectomy if surgical resection was evaluated to offer curative treatment.

### Data collection

Patients were identified from the archives using surgical registries and pathology reports. All relevant clinical data were retrospectively collected from electronic patient record systems used in the study hospitals. Tumour classification was updated to the American Joint Committee on Cancer (AJCC) 8^th^ edition of tumour-node-metastasis (TNM) classification [20]. Survival data until December 31, 2021, was received from Statistics Finland. The follow-up data were 100% complete.

Prospectively collected diagnostic H&E-stained histopathological slides of the primary tumours and pulmonary metastases were retrieved from pathology archives and reviewed by a pathologist. In pulmonary metastases, the most representative slide was selected for further analysis. In primary tumours, the slide with the deepest invasion depth was selected. The slides were digitalized with a × 20 objective magnification and resolution of 50,000 pixels per inch using an Aperio digital scanner AT2 Console (Leica Biosystems Imaging Inc., Wetzlar, Germany) or NanoZoomer-XR (Hamamatsu Photonics, Hamamatsu City, Japan).

### Histopathological examination

**Fig. 1:**
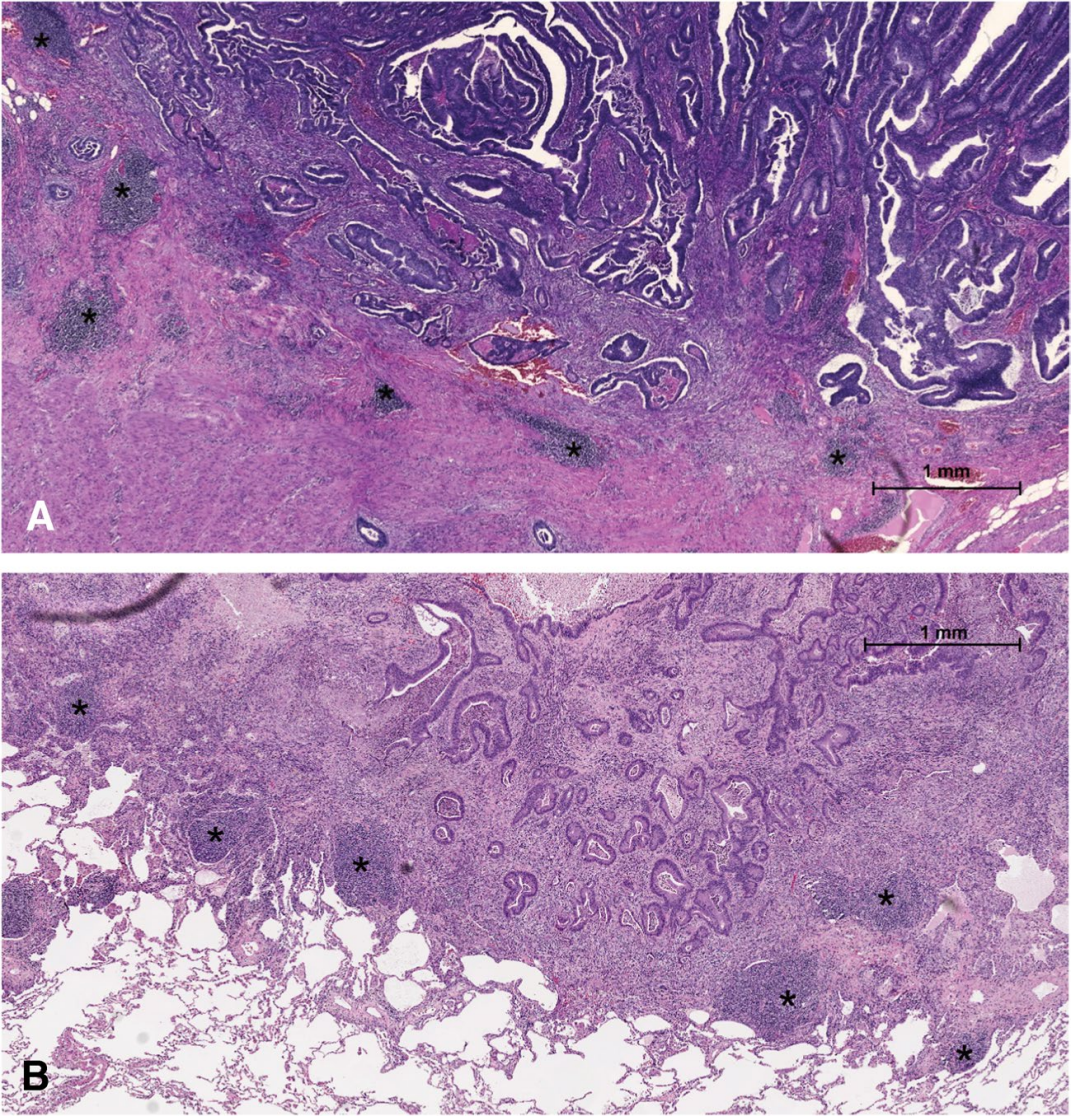
Haematoxylin and eosinophil–stained sections illustrating tertiary lymphoid structures (TLSs) in CRC primary tumours (**A**) and pulmonary metastases (**B**). Asterisks show examples of TLSs

A TLS was defined as a dense lymphocyte aggregate separate from the tumour bulk but within a 3.0-mm distance of the invasive margin of the tumour. Germinal centres were not a requirement. Perivascular lymphoid aggregates were also interpreted as TLSs. The minimum diameter accepted as a TLS was 150 µm. More diffuse peritumoural lymphocyte zones surrounding or in contact with the tumour bulk were not considered TLSs. TLS evaluation in a pulmonary metastasis and a primary tumour is illustrated in Fig. 1. The TLSs were assessed using three criteria. First, TLS density was calculated as the number of lymphocyte follicles divided by the length of the invasive margin as suggested by Väyrynen et al. [5]. Second, the diameter of the largest TLS was evaluated according to Ueno et al. [10]. Third, the number of TLSs in a hotspot was calculated using a field of view diameter of 5 mm.

Mismatch repair (MMR) and *BRAF* mutation status were determined by immunohistochemical analysis from the pulmonary metastases as described previously [17]. All patients were MMR proficient and 4.5% of all patients had a mutated *BRAF*.

### Tertiary lymphoid structure scoring

Receiver operating characteristics (ROC) analysis was used for optimal cut-off determination. The cut-off points for TLS density, TLS maximum diameter, and TLS hotspot count were 0.222 follicles/mm, 315 µm, and 3.50 follicles, respectively, in the pulmonary metastases (Supplementary Fig. 1) and 0.161 follicles/mm, 453 µm, and 3.50 follicles, respectively in the primary tumours (Supplementary Fig. 2).

For reproducibility assessment of the TLS evaluation in pulmonary metastases, two independent observers (T.Ka and J.P.V) independently conducted TLS evaluations on the first 20 pulmonary metastases. The interobserver agreement was measured as a continuous variable (Spearman *r*_*s*_) and a two-tiered variable (Cohen’s Kappa *κ*) using previously mentioned cut-offs. The interobserver agreement was excellent (TLS density *r*_*s*_ = 0.87, *κ* = 0.88; TLS maximum diameter *r*_*s*_ = 0.88, *κ* = 0.80; TLS hotspot count *r*_*s*_ = 0.93; *κ* = 0.88).

### Immune cell score

**Fig. 2:**
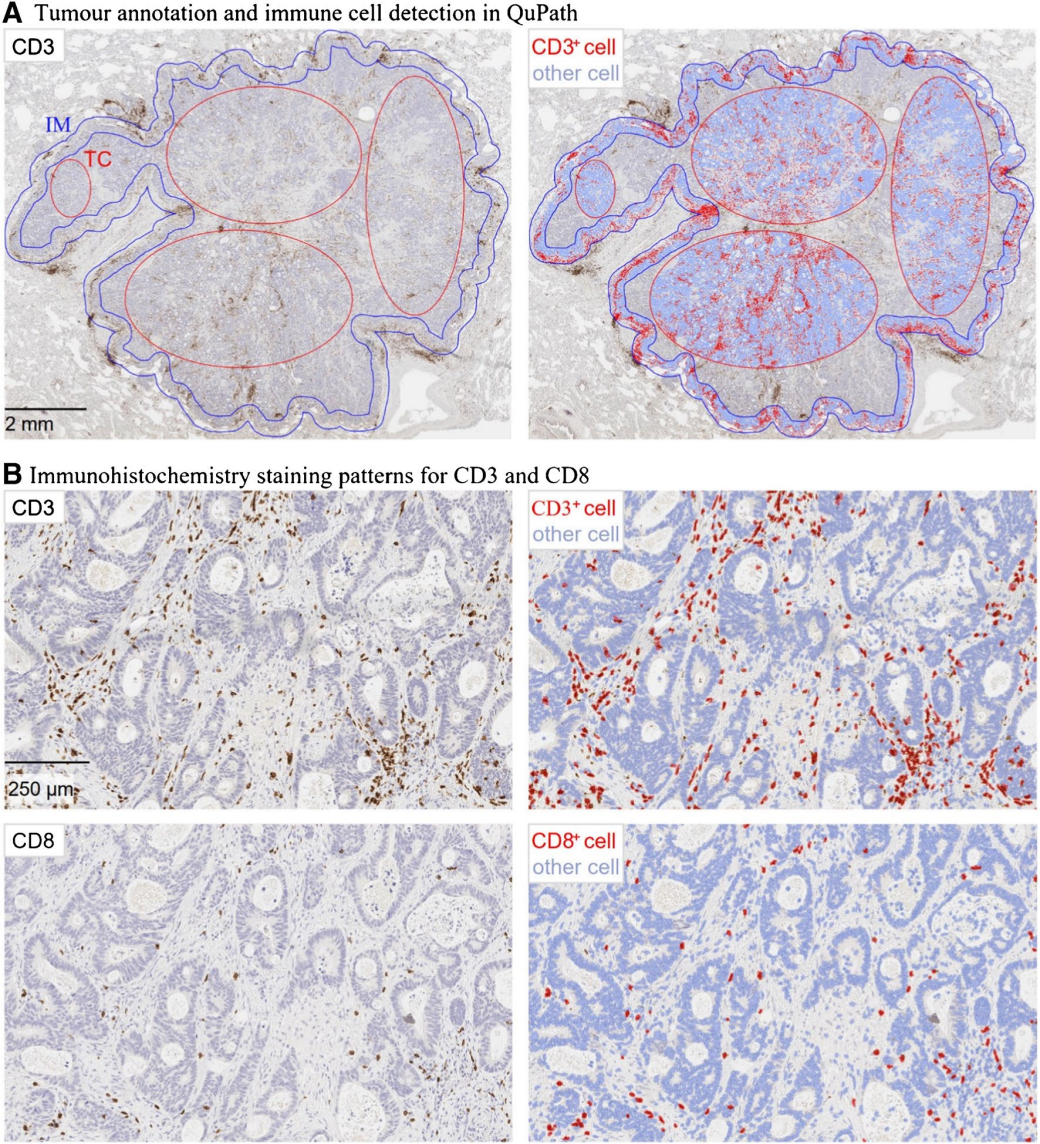
T-cell density analysis in a pulmonary metastasis of colorectal cancer. **A** Immune cell density analysis for representative sites of the tumour centre (TC) and the invasive margin (IM) using QuPath-bioimage software. The width of the invasive margin was 720 µm spanning 360 µm into the tumour and 360 μm into the healthy tissue. **B** Examples of CD3^+^ and CD8^+^ staining patterns and the positive cell detection in the respective site of the tumour

A CD3^+^ and CD8^+^ T-cell density-based ICS in the invasive margin and the tumour centre of the pulmonary metastases and primary CRC tumours was assessed as described earlier [17]. An example of an immune cell density analysis in a pulmonary metastasis is provided in Fig. 2. A three-tiered ICS classification was performed with predefined cut-offs of 25% and 70%, following the main principles of the consensus Immunoscore validation article [16]. An additional CD8^+^ T-cell density-based ICS for pulmonary metastases was evaluated following similar scoring methods as a sensitivity analysis.

### Outcomes and definitions

Royal College of Surgeons Charlson Score (RCS) was used for comorbidity classification [21]. The cancer under treatment was included as one comorbidity. Disease-free interval (DFI) was defined as the interval from surgery of CRC tumour to the date of detection of the first pulmonary metastasis. Pulmonary metastases that were detected less than 6 months after primary cancer treatment were deemed as synchronous and metachronous if otherwise.

The primary outcome of the study was 5-year overall survival from the date of pulmonary metastasectomy to death due to any cause before the end of follow-up. Only 1 patient died of another cause than cancer; therefore, cancer-specific survival was not analysed.

### Statistical analysis

The chi-square test or Fisher’s exact test was used for group comparison in categorical variables. For continuous variable comparison, a Student’s *t*-test was used for normally distributed variables and a Mann–Whitney *U*-test was used for continuous variables with a skewed distribution. Spearman correlation coefficients were used for bivariate correlation analysis. ROC analysis was used to determine optimal cut-offs using 5-year mortality after pulmonary metastasectomy and 10-year mortality after primary cancer surgery as state variables for pulmonary metastases and primary tumours, respectively. The cut-off value was determined as the point with the shortest distance from the coordinate (0,1).

In survival analysis, Kaplan–Meier survival curves were constructed from the first metastasectomy to death or end of the follow-up to visualize survival up to 5 years after pulmonary metastasectomy and log-rank tests to compare survivals. The estimates for hazard ratios (HR) with 95% confidence intervals (CI) were calculated using Cox proportional hazards regression. For multivariate analysis of TLS in pulmonary metastases, Cox regression models were adjusted for sex (female/male), age (continuous variable), RCS (1/ ≥ 2), neoadjuvant chemotherapy (no/yes), the synchronicity of pulmonary metastases (synchronous/metachronous), number of pulmonary metastases at diagnosis (1/ ≥ 2), and former liver metastasectomy (no/yes). For multivariate analysis in primary tumours, Cox regression models were adjusted for sex (female/male), age (continuous variable), RCS (1/ ≥ 2), neoadjuvant chemotherapy (no/yes), CRC stage (I–II/III/IV), and CRC grade (1/2/3). Additional adjusted models were constructed including ICS and each TLS measure in pulmonary metastases and primary tumours. Statistical analysis was performed using IBM SPSS Version 28 (IBM Corp., Armonk, NY, USA).

### Ethical aspects

The Oulu University Hospital Ethics Committee (EETMK 81/2008) approved the study. The Finnish National Authority of Medicolegal Affairs (VALVIRA) waived the need for informed consent due to the retrospective nature of the study. The study was performed in accordance with the Declaration of Helsinki.

## Results

### Patient characteristics

A total of 106 pulmonary metastasectomies from CRC were performed on 70 patients during the study period. Of the metastasectomies, 36 cases were re-metastasectomies performed on 21 patients. Adequate samples for TLS analysis were available for 100 pulmonary metastases and 63 primary tumours.

The final cohort of first pulmonary metastases and their corresponding primary CRCs consisted of 67 first resected pulmonary metastasis samples and 63 primary tumour samples. At the time of primary CRC treatment, five patients (7.1%) had stage I CRC, 18 patients (25.7%) had stage II CRC, 27 patients (38.6%) had stage III CRC, and 20 patients (28.6%) had stage IV CRC. The median DFI after primary CRC surgery was 337 (IQR 0–783) days. Twelve patients (17.1%) had bilateral pulmonary metastases and 35.7% of patients had more than one pulmonary metastasis. Four patients (5.7%) had an R1 resection of pulmonary metastases. Former liver metastases of CRC had been diagnosed and treated in 45.7% of all patients. Of all tumours, 50.0% were of rectal origin. The median follow-up time was 40.2 months (IQR 20.9–56.3), ranging from 5 to 233 months. The overall 5-year survival rate after pulmonary metastasectomy was 28.4%.

*BRAF* mutation status was not associated with overall survival after pulmonary metastasectomy or primary tumour resection.

### Tertiary lymphoid structures

The medians of TLS density, TLS maximum diameter, and TLS hotspot count in the first resected pulmonary metastases were 0.195 follicles/mm (IQR 0.054–0.362 follicles/mm), 325 µm (IQR 200–520 µm), and 3 follicles (IQR 1–5 follicles), respectively. Patient characteristics according to TLS density of metastases are presented in Table 1. Thirty-six patients had low TLS density (< 0.222 follicles/mm) and 31 patients had high TLS density (≥ 0.222 follicles/mm) in the first pulmonary metastases of CRC. TLS density of resected pulmonary metastases was not associated with neoadjuvant chemotherapy or other clinical baseline parameters.

**Table 1:** Patient characteristics (*n* = 67) according to the tertiary lymphoid structure (*TLS*) measures of the first pulmonary metastasis of colorectal cancer

	TLS density			TLS maximum diameter			TLS hotspot		
	Low	High	*p*-value	Low	High	*p*-value	Low	High	*p*-value
	*n* (%)	*n* (%)		*n* (%)	*n* (%)		*n* (%)	*n* (%)	
*n*	36	31		32	35		38	29	
Sex			0.067			0.389			0.067
Female	14 (38.9%)	19 (61.3%)		14 (43.8%)	19 (54.3%)		15 (39.5%)	18 (62.1%)	
Male	22 (61.1%)	12 (38.7%)		18 (56.3%)	16 (45.7%)		23 (60.5%)	11 (37.9%)	
Age (M; SD)	68.33 (9.70)	67 (11.15)	0.301	68.28 (9.98)	67.2 (10.77)	0.336	69.39 (9.37)	65.52 (11.27)	0.065
RCS			0.842			0.444			0.954
1	21 (58.3%)	20 (64.5%)		21 (65.6%)	20 (57.1%)		23 (60.5%)	18 (62.1%)	
2	9 (25.0%)	6 (19.4%)		5 (15.6%)	10 (28.6%)		9 (23.7%)	6 (20.7%)	
≥ 3	6 (16.7%)	5 (16.1%)		6 (18.8%)	5 (14.3%)		6 (15.8%)	5 (17.2%)	
Neoadjuvant chemotherapy			0.331			0.072			0.952
No	19 (52.8%)	20 (64.5%)		15 (46.9%)	24 (68.6%)		22 (57.9%)	17 (58.6%)	
Yes	17 (47.2%)	11 (35.5%)		17 (53.1%)	11 (31.4%)		16 (42.1%)	12 (41.4%)	
CRC stage			0.597			0.321			0.771
1–2	11 (30.6%)	12 (38.7%)		13 (40.6%)	10 (28.6%)		12 (31.6%)	11 (37.9%)	
3	13 (36.1%)	12 (38.7%)		9 (28.1%)	16 (45.7%)		14 (36.8%)	11 (37.9%)	
4	12 (33.3%)	7 (22.6%)		10 (31.3%)	9 (25.7%)		12 (31.6%)	7 (24.1%)	
Primary tumour location			0.895			0.273			0.724
Colon	18 (50.0%)	16 (51.6%)		14 (43.8%)	20 (57.1%)		20 (52.6%)	14 (48.3%)	
Rectum	18 (50.0%)	15 (48.4%)		18 (56.3%)	15 (42.9%)		18 (47.4%)	15 (51.7%)	
Former LM			0.558			0.726			0.941
No	20 (55.6%)	15 (48.4%)		16 (50.0%)	19 (54.3%)		20 (52.6%)	15 (51.7%)	
Yes	16 (44.4%)	16 (51.6%)		16 (50.0%)	16 (45.7%)		18 (47.4%)	14 (48.3%)	
DFI (MD, IQR)	288 (0–843.5)	427 (0–773)	0.569	325 (0–703.5)	482 (0–813)	0.417	288 (0–750)	482 (0–813)	0.351
No. of PM			0.627			0.239			0.977
1	21 (58.3%)	22 (71.0%)		18 (56.3%)	25 (71.4%)		24 (63.2%)	19 (65.5%)	
2	12 (33.3%)	7 (22.6%)		10 (31.3%)	9 (25.7%)		11 (28.9%)	8 (27.6%)	
≥ 3	3 (8.3%)	2 (6.5%)		4 (12.5%)	1 (2.9%)		3 (7.9%)	2 (6.9%)	
Laterality of metastases			0.321			0.864			0.443
Unilateral	28 (77.8%)	27 (87.1%)		26 (81.3%)	29 (82.9%)		30 (78.9%)	25 (86.2%)	
Bilateral	8 (22.2%)	4 (12.9%)		6 (18.8%)	6 (17.1%)		8 (21.1%)	4 (13.8%)	
Synchronicity			0.773			0.85			0.971
Synchronous	8 (22.2%)	6 (19.4%)		7 (21.9%)	7 (20.0%)		8 (21.1%)	6 (20.7%)	
Metachronous	28 (77.8%)	25 (80.6%)		25 (78.1%)	28 (80.0%)		30 (78.9%)	23 (79.3%)	
Size of largest PM (cm; MD; IQR)	1.5 (1–3.5)	2.2 (1.5–3.5)	0.303	2 (1.1–3.5)	2 (1.3–3.5)	0.746	2 (1–3.5)	2.2 (1.5–3.1)	0.376
ICS of metastases			0.031*			0.009*			0.24
Low	7 (19.4%)	2 (6.9%)		6 (18.8%)	3 (9.1%)		6 (15.8%)	3 (11.1%)	
Intermediate	24 (66.7%)	15 (51.7%)		23 (71.9%)	16 (48.5%)		25 (65.8%)	14 (51.9%)	
High	5 (13.9%)	12 (41.4%)		3 (9.4%)	14 (42.4%)		7 (18.4%)	10 (37.0%)	
ICS of primary tumour			0.004*			0.003*			0.221
Low	10 (29.4%)	2 (8.0%)		8 (29.6%)	4 (12.5%)		9 (25.0%)	3 (13.0%)	
Intermediate	21 (61.8%)	12 (48.0%)		18 (66.7%)	15 (46.9%)		21 (58.3%)	12 (52.2%)	
High	3 (8.8%)	11 (44.0%)		1 (3.7%)	13 (40.6%)		6 (16.7%)	8 (34.8%)	
prim TLS density			0.053			0.069			0.04*
Low	18 (58.1%)	9 (30.0%)		15 (57.7%)	12 (34.3%)		19 (55.9%)	8 (29.6%)	
High	13 (41.9%)	21 (70.0%)		11 (42.3%)	23 (65.7%)		15 (44.1%)	19 (70.4%)	
prim TLS max diameter			0.054			0.007*			0.142
Low	23 (74.2%)	17 (56.7%)		22 (84.6%)	18 (51.4%)		25 (73.5%)	15 (55.6%)	
High	8 (25.8%)	13 (43.3%)		4 (15.4%)	17 (48.6%)		9 (26.5%)	12 (44.4%)	
prim TLS hotspot			0.249			0.087			0.21
Low	21 (67.7%)	16 (53.3%)		19 (73.1%)	18 (51.4%)		23 (67.6%)	14 (51.9%)	
High	10 (32.3%)	14 (46.7%)		7 (26.9%)	17 (48.6%)		11 (32.4%)	13 (48.1%)	

The variables were dichotomized using cut-offs selected based on the receiver operating characteristics analysis

*RCS* Royal College of Surgeons Charlson Score, *CRC* colorectal carcinoma, *DFI* disease-free interval, *ICS* immune cell score, *LM* liver metastasectomy, *PM* pulmonary metastases, *prim* primary tumour

^*^Statistically significant at the level of < 0.05

In primary tumours, the medians of TLS density, TLS maximum diameter, and TLS hotspot count were 0.195 follicles/mm (IQR 0.073–0.418 follicles/mm), 383 µm (IQR 260–577 µm), and 3 follicles (IQR 1–5 follicles), respectively. Thirty patients had low TLS density (≤ 0.161 follicles/mm) and 33 patients high TLS density (> 0.161 follicles/mm). Patient characteristics according to TLSs of the primary tumour are presented in Supplementary table 1. Higher TLS densities were more uncommon in rectal cancer (*p* = 0.009) and rarely associated with synchronous pulmonary metastases, although the difference was not statistically significant (*p* = 0.051). Patients who received neoadjuvant chemotherapy for primary tumours had significantly lower TLS density, maximum diameter, and hotspot density in primary tumours (Supplementary table 1).

There was no statistically significant difference in the median TLS measures between the first resected pulmonary metastases and primary tumours using the three criteria (TLS density 0.195 vs. 0.195 follicles/mm, *p* = 0.791; TLS maximum diameter 325 vs. 383 µm, *p* = 0.158; TLS hotspot 3 vs. 3 follicles, *p* = 0.725). TLS assessments with the three different criteria were significantly correlated with each other both in pulmonary metastases and primary tumours (*r*_*s*_ = 0.79–0.95, *p* < 0.001 in pulmonary metastases; *r*_*s*_ = 0.717–0.887, *p* < 0.001 in primary tumours; Table 2). TLS measures between the first pulmonary metastases and primary tumours were not significantly correlated in continuous variable analysis. However, after dichotomization using cut-off values selected using the ROC analysis, TLS measures in the pulmonary metastases were associated with those of the primary tumour (TLS maximum diameter: *p* = 0.007; Table 1).

**Table 2:** Spearman correlation analysis of tertiary lymphoid structures (TLS) in the first resected pulmonary metastases of colorectal cancer and primary tumours

			Pulmonary metastases			Primary tumour		
			TLS density	TLS max diameter	TLS hotspot	TLS density	TLS max diameter	TLS hotspot
Pulmonary metastases	TLS density	*r*_*s*_	1.000	0.815**	0.949**	0.128	0.096	0.164
		*p*		< 0.001	< 0.001	0.327	0.461	0.207
		*N*	67	67	67	61	61	61
	TLS max diameter	*r*_*s*_	0.815**	1.000	0.778**	0.130	0.144	0.131
		*p*	< 0.001		< 0.001	0.320	0.267	0.313
		*N*	67	67	67	61	61	61
	TLS hotspot	*r*_*s*_	0.949**	0.778**	1.000	0.098	0.100	0.123
		*p*	< 0.001	< 0.001		0.451	0.444	0.343
		*N*	67	67	67	61	61	61
Primary tumour	TLS density	*r*_*s*_	0.134	0.136	0.098	1.000	0.781**	0.887**
		*p*	0.302	0.297	0.451		< 0.001	< 0.001
		*N*	61	61	61	63	63	63
	TLS max diameter	*r*_*s*_	0.096	0.144	0.100	0.781**	1.000	0.717**
		*p*	0.461	0.267	0.444	< 0.001		< 0.001
		*N*	61	61	61	63	63	63
	TLS hotspot	*r*_*s*_	0.164	0.131	0.123	0.887**	0.717**	1.000
		*p*	0.207	0.313	0.343	< 0.001	< 0.001	
		*N*	61	61	61	63	63	63

*r*_*s*_, Spearman’s rank correlation coefficient

^**^Correlation is significant at the 0.01 level

### Tertiary lymphoid structures and survival

**Fig. 3:**
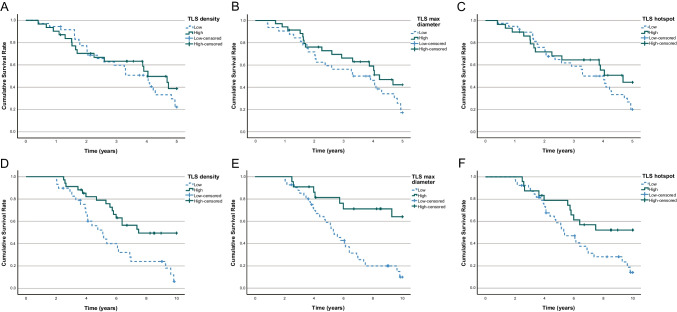
K-M curves of 5-year overall survival after pulmonary metastasectomy according to **A** tertiary lymphoid structure (TLS) density (*p* = 0.405), **B** TLS maximum diameter (*p* = 0.118), **C** TLS hotspot count (*p* = 0.209) in the pulmonary metastases, and **D** TLS density (*p* = 0.002), **E** TLS maximum diameter (*p* < 0.001), and **F** the TLS hotspot count (*p* = 0.02) in the primary tumours. Log-rank tests were applied

TLSs in the first resected pulmonary metastases had no statistically significant effect on 5-year survival in K-M analysis (TLS density low 22.2% vs. high 38.7%, *p* = 0.405; TLS maximum diameter low 17.1% vs. high 42.2%, *p* = 0.118; TLS hotspot low 20.0% vs. high 44.4%, *p* = 0.209; Fig. 3A–C). In multivariable analysis, TLS did not affect 5-year overall survival (Table 3).

**Table 3:** Hazard ratios (*HR*) for 5-year all-cause mortality with 95% confidence intervals in the first pulmonary metastases and primary colorectal tumours according to tertiary lymphoid structure (TLS) measures

		TLS density		TLS maximum diameter		TLS hotspot	
	*n*	Low, HR (95% CI)	High, HR (95% CI)	Low, HR (95% CI)	High, HR (95% CI)	Low, HR (95% CI)	High, HR (95% CI)
Metastases							
Crude	67	1.00 (reference)	0.77 (0.41–1.44; *p* = 0.407)	1.00 (reference)	0.61 (0.33–1.14; *p* = 0.122)	1.00 (reference)	0.66 (0.35–1.27; *p* = 0.212)
Adjusted*	67	1.00 (reference)	0.91 (0.48–1.73; *p* = 0.763)	1.00 (reference)	0.78 (0.40–1.51; *p* = 0.458)	1.00 (reference)	0.90 (0.50–1.80; *p* = 0.763)
Primary tumours							
Crude	58	1.00 (reference)	0.38 (0.20–0.72, *p* = 0.003)	1.00 (reference)	0.25 (0.11–0.57; *p* < 0.001)	1.00 (reference)	0.44 (0.2–0.90; *p* = 0.024)
Adjusted**	58	1.00 (reference)	0.39 (0.18–0.87; *p* = 0.022)	1.00 (reference)	0.28 (0.11–0.73; *p* = 0.01)	1.00 (reference)	0.51 (0.22–1.17; *p* = 0.112)

*TLS*, tertiary lymphoid structure

^*^Adjusted for sex (female/male), age (continuous), RCS (1/ ≥ 2), neoadjuvant chemotherapy (no/yes), synchronicity of pulmonary metastases (synchronous/metachronous), number of pulmonary metastases at diagnosis (1/ ≥ 2), former liver metastasectomy (no/yes)

^**^Adjusted for sex (female/male), age (continuous), RCS (1/ ≥ 2), neoadjuvant chemotherapy (no/yes), CRC stage (I–II/III/IV), CRC grade (1/2/3)

TLSs in primary tumours were associated with longer survival in 10-year K-M survival analysis (TLS density low 6.0% vs. high 49.4%, *p* < 0.001; TLS maximum diameter low 10.0% vs. high 64.1%, *p* < 0.001; TLS hotspot low 14.1% vs. high 52.3%, *p* = 0.02; Fig. 3D–F). In multivariate analysis, TLS density and maximum diameter had a statistically significant effect on 10-year overall survival (TLS density adjusted HR 0.39, 0.18–0.87, *p* = 0.022; TLS maximum diameter adjusted HR 0.28, 0.11–0.73, *p* = 0.01; Table 3).

### Tertiary lymphoid structures and immune cell score

In the first resected pulmonary metastases, ICS was significantly associated with TLS density (*p* = 0.031) and the maximum diameter of TLS (*p* = 0.009, Table 1). In continuous variable correlation analysis, the CD8^+^ density in the invasive margin of the pulmonary metastases had a moderate to strong correlation with all the TLS measures (TLS density *r*_*s*_ = 0.368, *p* = 0.003; TLS maximum diameter *r*_*s*_ =  − 0.481, *p* < 0.001; TLS hotspot *r*_*s*_ = 0.333, *p* = 0.007; Table 4). Additionally, the TLS maximum diameter significantly correlated with CD8^+^ density in the tumour centre and CD3^+^ density in the invasive margin of pulmonary metastases. In the primary tumour, TLS measures and CD3^+^ and CD8^+^ cell densities were not significantly correlated in continuous or categorical variable analysis (Table 4 and Supplementary table 1).

**Table 4:** Spearman correlations of tertiary lymphoid structures (*TLSs*) and CD3^+^ and CD8^+^ T-cell densities in the first pulmonary metastases of colorectal cancer and primary tumours

			CD3 + (TC)	CD3 + (IM)	CD8 + (TC)	CD8 + (IM)
Metastases	TLS density	*r*_*s*_	0.167	0.241	0.242	0.368**
		*p*	0.183	0.055	0.052	0.003
		*N*	65	64	65	64
	TLS max diameter	*r*_*s*_	0.176	0.264*	0.315*	0.481**
		*p*	0.162	0.035	0.011	< 0.001
		*N*	65	64	65	64
	TLS hotspot	*r*_*s*_	0.110	0.205	0.202	.333**
		*p*	0.382	0.103	0.107	0.007
		*N*	65	64	65	64
Primary tumour	TLS density	*r*_*s*_	0.096	0.147	0.221	0.220
		*p*	0.472	0.269	0.095	0.100
		*N*	58	58	58	57
	TLS max diameter	*r*_*s*_	0.011	0.208	0.139	0.216
		*p*	0.937	0.118	0.299	0.107
		*N*	58	58	58	57
	TLS hotspot	*r*_*s*_	0.134	0.160	0.232	0.208
		*p*	0.317	0.231	0.080	0.121
		*N*	58	58	58	57

*IM* invasive margin, *r*_*s*_ Spearman’s rank correlation co-efficient, *TC* tumour centre

^**^Correlation is significant at the 0.01 level

^*^Correlation is significant at the 0.05 level

In the survival analysis using Cox regression models including TLS and ICS, TLS measures did not have prognostic value in the first resected pulmonary metastases, whereas low ICS was significantly associated with worse survival compared to intermediate ISC (model 2: adjusted HR 0.33, 0.13–0.81, *p* = 0.016; Supplementary table 2) and high ICS (model 2: adjusted HR 0.22, 0.06–0.78, *p* = 0.019; Supplementary table 2). In the primary tumour, ICS did not have prognostic value, whereas TLS had significant to suggestive prognostic value (low vs. high TLS density: adjusted HR 0.43, 0.18–1.02, *p* = 0.055; low vs. high TLS maximum diameter: adjusted HR 0.31, 0.11–0.84, *p* = 0.021; low vs. high TLS hotspot: adjusted HR 0.50, 0.20–1.25, *p* = 0.139; Supplementary table 2). In further analysis, a CD8^+^ cell density-based three-tiered ICS in pulmonary metastases had a significant effect on 5-year overall survival (low 9.1%, intermediate 24.1%, high 47.3%, *p* = 0.029; Supplementary Fig. 3).

## Discussion

We performed a study on the prognostic effect of TLSs in resected pulmonary metastases of CRC and the corresponding primary tumours. The main finding of this study is that TLS, assessed using H&E-stained slides, has a significant survival effect in primary tumours but not in the first resected pulmonary metastases. TLS maximum diameters in the pulmonary metastases were positively associated with those of the primary tumours.

There is no clear consensus on the definition of a TLS. Some authors suggest TLSs to be defined rather strictly with germinal centres and certain lymphoid cell composition and cytokines [22], while other authors suggest also small lymphoid cell clusters to be included in the definition and interpreted as immature TLSs, as part of a continuum of TLSs [3]. More rigorous definitions of TLSs require immunohistochemistry, whereas TLSs defined as lymphocyte aggregates could be assessed using H&E-stained slides in routine diagnostics. By using H&E staining to assess TLSs, several studies of primary colorectal tumours have proven that low CLR is a risk factor for cancer progression with poor cancer-specific and overall survival [5, 8, 10, 23]. In metastatic CRC, a few studies have demonstrated TLSs as a prognosticator in liver and pulmonary metastases; however, they are mainly based on immunohistochemical analysis of single cell types [14]. We found one previous study evaluating TLSs in H&E-stained sections of liver metastases, only without survival analysis [24]. In our study, TLSs in the pulmonary metastases had no survival effect in univariable or multivariable analysis. In multivariate models including ICS and TLS measures in pulmonary metastases, ICS had significant prognostic value irrespective of TLSs, and TLSs had no prognostic value. However, there was significant association between the H&E-staining–based TLS measures and the CD3^+^ and CD8^+^ densities and ICS in the pulmonary metastases, suggesting that TLS measures might be used in screening patients for high T-cell infiltration and ICS. Due to the strong correlation between TLS measures and especially CD8^+^ cell densities in the pulmonary metastases, we constructed an additional CD8^+^ cell density-based ICS, which also had a significant survival association in 5-year follow-up. There was also an association between primary tumours and pulmonary metastases: TLS measures in the first pulmonary metastases were associated with the CD3^+^ and CD8^+^ cell densities and ICS of the primary tumours. Correlation between immune infiltrates in primary tumours and matched lung metastases has been seen previously [25]. The lack of prognostic value of TLSs in the pulmonary metastases in our study might be partly explained by selection bias, as patients with the most aggressive and disseminated diseases and possibly milder immune responses presumably end up excluded from surgical pulmonary metastasectomy. On the other hand, this result might reflect the shifted immune contexture in the pulmonary metastases, contrasting the primary tumours. Still, the sample size was relatively small and presented confidence intervals wide including clinically significant HRs. Further studies are needed to determine the prognostic value of TLSs in CRC pulmonary metastases.

Concerning the primary colorectal tumours in our study, high TLS measures in the primary tumours had a significant positive effect on 10-year survival in univariable and multivariable analyses. This result is concordant with reference literature [5, 9, 10]. Considering the obvious selection bias of primary tumours in our study, the significant prognostic value of TLS, but not ICS, in the primary tumours is interesting and underlines the significance of TLSs/CLR in primary tumours irrespective of Immunoscore, which has proven prognostic value validated in large international studies [16]. The difference between the primary tumours and pulmonary metastases in the prognostic value of TLSs and ISC is intriguing. With respect to genetic mutations, a previous study suggested that the prognostic value of CLR is limited to pMMR CRC [26]. All our patients were pMMR, which might account for our significant results in primary tumours. The proportion of dMMR cases among metastatic colorectal cancer cases is much lower than in primary CRC of all stages, around 4–5% [27], and it appears even lower for CRC patients with pulmonary metastases [28]. Neoadjuvant chemotherapy of the primary tumour was also associated with the TLS measures of the primary tumour: patients not receiving neoadjuvant chemotherapy had significantly higher TLS measures. Although we cannot exclude the possibility of this affecting the survival analysis results, we took it into consideration by adjusting the multivariable survival models for neoadjuvant chemotherapy status. Interestingly, a similar effect was not seen in the pulmonary metastases, which might also illustrate the shifted immune milieu in the metastases of CRC.

The novelty of this study can be considered as a strength; to the best of our knowledge, there are no previous studies on the prognostic value of TLSs assessed from H&E-stained slides of CRC metastases. This is a dual-institutional study, which also can be considered as a strength from a clinical point of view. As a population-based study, the selection bias is apparent when restricting to surgically managed patients only, and the results cannot be generalized to most patients with pulmonary metastases, as most patients are not treated operatively. The relatively small sample size is a limitation in our study. It naturally results in a long study period which might produce confounding due to improvements in therapy and diagnostics during the study period.

## Conclusion

Despite the significant association between TLS measures in the first pulmonary metastases and primary tumours and the significant prognostic value of TLSs in primary tumours, TLSs had no prognostic value in the resected pulmonary metastases of CRC. In the pulmonary metastases, ICS had superior prognostic value to the TLS measures, while, in primary tumours, TLS measures independently predicted survival.

## Supplementary Information

Below is the link to the electronic supplementary material.

Supplementary file1 (DOCX 283 KB)

## Data Availability

Data is available from the corresponding author upon reasonable request. Data sharing will require an additional ethical board statement.

## References

[1] Gonzalez H, Hagerling C, Werb Z (2018). Roles of the immune system in cancer: from tumor initiation to metastatic progression. Genes Dev.

[2] Pitzalis C, Jones GW, Bombardieri M, Jones SA (2014). Ectopic lymphoid-like structures in infection, cancer and autoimmunity. Nat Rev Immunol.

[3] Colbeck EJ, Ager A, Gallimore A, Jones GW (2017) Tertiary lymphoid structures in cancer: drivers of antitumor immunity, immunosuppression, or bystander sentinels in disease? Front Immunol 8:1830 10.3389/fimmu.2017.01830 PMC5742143 29312327

[4] Dieu-Nosjean MC, Antoine M, Danel C, Heudes D, Wislez M, Poulot V (2008). Long-term survival for patients with non-small-cell lung cancer with intratumoral lymphoid structures. J Clin Oncol.

[5] Väyrynen JP, Sajanti SA, Klintrup K, Mäkelä J, Herzig KH, Karttunen TJ (2014). Characteristics and significance of colorectal cancer associated lymphoid reaction. Int J Cancer.

[6] Zhang NN, Qu FJ, Liu H, Li ZJ, Zhang YC, Han X et al (2021) Prognostic impact of tertiary lymphoid structures in breast cancer prognosis: a systematic review and meta-analysis. Cancer Cell Int 21(1):536–546 10.1186/s12935-021-02242-x PMC8520238 34654433

[7] Sung H, Ferlay J, Siegel R, Laversanne M, Soerjomataram I, Jemal A (2020). Global Cancer Statistics 2020: GLOBOCAN estimates of incidence and mortality worldwide for 36 cancers in 185 countries. CA Cancer J Clin.

[8] Graham D, Appelman H (1990). Crohn’s-like lymphoid reaction and colorectal carcinoma: a potential histologic prognosticator. Mod Pathol.

[9] Maoz A, Dennis M, Greenson JK (2019) The Crohn’s-like lymphoid reaction to colorectal cancer-tertiary lymphoid structures with immunologic and potentially therapeutic relevance in colorectal cancer. Front Immunol 10:1884 10.3389/fimmu.2019.01884 PMC6714555 31507584

[10] Ueno H, Hashiguchi Y, Shimazaki H, Shinto E, Kajiwara Y, Nakanishi K (2013). Objective criteria for crohn-like lymphoid reaction in colorectal cancer. Am J Clin Pathol.

[11] Mitry E, Guiu B, Cosconea S, Jooste V, Faivre J, Bouvier AM (2010). Epidemiology, management and prognosis of colorectal cancer with lung metastases: a 30-year population-based study. Gut.

[12] Väyrynen V, Wirta EV, Seppälä T, Sihvo E, Mecklin JP, Vasala K (2020). Incidence and management of patients with colorectal cancer and synchronous and metachronous colorectal metastases: a population-based study. BJS Open.

[13] Colorectal Cancer Survival Rates | Colorectal Cancer Prognosis [Internet]. Available from: https://www.cancer.org/cancer/colon-rectal-cancer/detection-diagnosis-staging/survival-rates.html. Accessed 20 Sept 2022

[14] Schweiger T, Berghoff AS, Glogner C, Glueck O, Rajky O, Traxler D (2016). Tumor-infiltrating lymphocyte subsets and tertiary lymphoid structures in pulmonary metastases from colorectal cancer. Clin Exp Metastasis.

[15] Ahmed A, Halama N (2020) Tertiary lymphoid structures in colorectal cancer liver metastases: association with immunological and clinical parameters and chemotherapy response. Anticancer Res 40(11):6367–6373 10.21873/anticanres.14657 33109574

[16] Pagès F, Mlecnik B, Marliot F, Bindea G, Ou FS, Bifulco C (2018). International validation of the consensus Immunoscore for the classification of colon cancer: a prognostic and accuracy study. Lancet.

[17] Karjula T, Elomaa H, Niskakangas A, Mustonen O, Puro I, Kuopio T (2023). CD3+ and CD8+ T-cell-based immune cell score and PD-(L)1 expression in pulmonary metastases of microsatellite stable colorectal cancer. Cancers (Basel).

[18] Yun W, Hao-cheng L, Ma-yan H, Qiong S, Zhi-qiang W, Feng-hua W (2018). The Immunoscore system predicts prognosis after liver metastasectomy in colorectal cancer liver metastases. Cancer Immunol.

[19] Lea D, Haland S, Hagland HR, Soreide K (2014). Accuracy of TNM staging in colorectal cancer: a review of current culprits, the modern role of morphology and stepping-stones for improvements in the molecular era. Scand J Gastroenterol.

[20] Amin MB, Edge S, Greene F, Byrd DR, Brookland RK, Washington MK (2017). AJCC cancer staging manual.

[21] Armitage JN, van der Meulen JH (2010). Identifying co-morbidity in surgical patients using administrative data with the Royal College of Surgeons Charlson Score. Br J Surg.

[22] Dieu-Nosjean MC, Goc J, Giraldo NA, Sautès-Fridman C, Fridman WH (2014). Tertiary lymphoid structures in cancer and beyond. Trends Immunol.

[23] Ogino S, Nosho K, Irahara N, Meyerhardt JA, Baba Y, Shima K (2009). Lymphocytic reaction to colorectal cancer is associated with longer survival, independent of lymph node count, MSI and CpG island methylator phenotype. Clin Cancer Res.

[24] Höppener DJ, Stook JLPL, Galjart B, Nierop PMH, Nagtegaal ID, Vermeulen PB et al (2022) The relationship between primary colorectal cancer histology and the histopathological growth patterns of corresponding liver metastases. BMC Cancer 22(1):911–926 10.1186/s12885-022-09994-3 PMC9394040 35996090

[25] Ahtiainen M, Elomaa H, Väyrynen JP, Wirta EV, Kuopio T, Helminen O (2021). Immune contexture of MMR-proficient primary colorectal cancer and matched liver and lung metastases. Cancers (Basel).

[26] Rozek LS, Schmit SL, Greenson JK, Tomsho LP, Rennert HS, Rennert G et al (2016) Tumor-infiltrating lymphocytes, Crohn’s-like lymphoid reaction, and survival from colorectal cancer. JNCI J Natl Cancer Inst 108(8):djw027 10.1093/jnci/djw027 PMC5017930 27172903

[27] Buchler T (2022) Microsatellite instability and metastatic colorectal cancer - a clinical perspective. Front Oncol 12:888181 10.3389/fonc.2022.888181 PMC9097548 35574322

[28] Melloni G, Doglioni C, Bandiera A, Carretta A, Ciriaco P, Arrigoni G (2006). Prognostic factors and analysis of microsatellite instability in resected pulmonary metastases from colorectal carcinoma. Ann Thorac Surg.

