# Supplementary material for: Tertiary lymphoid structures in pulmonary metastases of microsatellite stable colorectal cancer
Source: Virchows Arch. 2023 Jun 20;483(1):21–32. doi: 10.1007/s00428-023-03577-8 (PMC10326136; doi:10.1007/s00428-023-03577-8)
Supplement: Supplementary file 1 — Supplementary file1 (DOCX 283 KB) [file 428_2023_3577_MOESM1_ESM.docx]

**Supplementary Material**

**Supplementary table 1.** Patient characteristics according to tertiary lymphoid structures (TLSs) in the primary tumours.

|  | TLS density | | | TLS max diameter | | | TLS hotspot | | |
| --- | --- | --- | --- | --- | --- | --- | --- | --- | --- |
|  | Low | High | p-value | Low | High | p-value | Low | High | p-value |
|  | n (%) | n (%) |  | n (%) | n (%) |  | n (%) | n (%) |  |
| n | 30 | 33 |  | 31 | 32 |  | 39 | 24 |  |
| Sex |  |  | 0.891 |  |  | 0.663 |  |  | 0.921 |
| Female | 15 (51.7%) | 17 (50.0%) |  | 20 (48.8%) | 12 (54.5%) |  | 20 (51.3%) | 12 (50.0%) |  |
| Male | 14 (48.3%) | 17 (50.0%) |  | 21 (51.2%) | 10 (45.5%) |  | 19 (48.7%) | 12 (50.0%) |  |
| Age (M; SD) | 65.8 (11.43) | 68.7 (9.66) | 0.138 | 67.7 (10.79) | 66.6 (10.23) | 0.352 | 66.6 (10.72) | 68.5 (10.33) | 0.255 |
| RCS |  |  | 0.141 |  |  | **0.012*** |  |  | 0.111 |
| 1 | 19 (65.5%) | 20 (58.8%) |  | 29 (70.7%) | 10 (45.5%) |  | 26 (66.7%) | 13 (54.2%) |  |
| 2 | 4 (13.8%) | 11 (32.4%) |  | 5 (12.2%) | 10 (45.5%) |  | 6 (15.4%) | 9 (37.5%) |  |
| ≥3 | 6 (20.7%) | 3 (8.8%) |  | 7 (17.1%) | 2 (9.1%) |  | 7 (17.9%) | 2 (8.3%) |  |
| Neoadjuvant chemotherapy |  |  | **<0.001*** |  |  | **0.026*** |  |  | **0.018*** |
| No | 15 (51.7%) | 31 (93.9%) |  | 26 (65.0%) | 20 (90.9%) |  | 25 (64.1%) | 21 (91.3%) |  |
| Yes | 14 (48.3%) | 2 (6.1%) |  | 14 (35.0%) | 2 (9.1%) |  | 14 (35.9%) | 2 (8.7%) |  |
| CRC stage |  |  | 0.106 |  |  | 0.106 |  |  | 0.373 |
| 1-2 | 6 (20.7%) | 15 (44.1%) |  | 14 (34.1%) | 7 (31.8%) |  | 11 (28.2%) | 10 (41.7%) |  |
| 3 | 13 (44.8%) | 13 (38.2%) |  | 14 (34.1%) | 12 (54.5%) |  | 16 (41.0%) | 10 (41.7%) |  |
| 4 | 10 (34.5%) | 6 (17.6%) |  | 13 (31.7%) | 3 (13.6%) |  | 12 (30.8%) | 4 (16.7%) |  |
| Grade |  |  | 0.312 |  |  | 0.607 |  |  | 0.291 |
| 1 | 5 (17.9%) | 11 (35.5%) |  | 12 (30.0%) | 4 (21.1%) |  | 9 (24.3%) | 7 (31.8%) |  |
| 2 | 18 (64.3%) | 16 (51.6%) |  | 23 (57.5%) | 11 (57.9%) |  | 24 (64.9%) | 10 (45.5%) |  |
| 3 | 5 (17.9%) | 4 (12.9%) |  | 5 (12.5%) | 4 (21.1%) |  | 4 (10.8%) | 5 (22.7%) |  |
| Tumour location |  |  | **0.009*** |  |  | 0.19 |  |  | 0.458 |
| Colon | 10 (34.5%) | 23 (67.6%) |  | 19 (46.3%) | 14 (63.6%) |  | 19 (48.7%) | 14 (58.3%) |  |
| Rectum | 19 (65.5%) | 11 (32.4%) |  | 22 (53.7%) | 8 (36.4%) |  | 20 (51.3%) | 10 (41.7%) |  |
| DFI (d; MD; IQR) | 188 (0–750) | 498 (259–773) | 0.123 | 338 (0–745) | 567 (259–1007) | 0.097 | 309 (0–750) | 454.5 (255–1001) | 0.163 |
| Laterality of PM |  |  | 0.533 |  |  | 0.558 |  |  | 0.896 |
| Unilateral | 23 (79.3%) | 29 (85.3%) |  | 33 (80.5%) | 19 (86.4%) |  | 32 (82.1%) | 20 (83.3%) |  |
| Bilateral | 6 (20.7%) | 5 (14.7%) |  | 8 (19.5%) | 3 (13.6%) |  | 7 (17.9%) | 4 (16.7%) |  |
| Synchronicity |  |  | 0.051 |  |  | 0.912 |  |  | 0.416 |
| Synchronous | 8 (27.6%) | 3 (8.8%) |  | 7 (17.1%) | 4 (18.2%) |  | 8 (20.5%) | 3 (12.5%) |  |
| Metachronous | 21 (72.4%) | 31 (91.2%) |  | 34 (82.9%) | 18 (81.8%) |  | 31 (79.5%) | 21 (87.5%) |  |
| ICS of primary tumour |  |  | 0.469 |  |  | 0.315 |  |  | 0.159 |
| 0 | 8 (29.6%) | 5 (16.1%) |  | 10 (26.3%) | 3 (15.0%) |  | 11 (29.7%) | 2 (9.5%) |  |
| 1 | 13 (48.1%) | 18 (58.1%) |  | 21 (55.3%) | 10 (50.0%) |  | 19 (51.4%) | 12 (57.1%) |  |
| 2 | 6 (22.2%) | 8 (25.8%) |  | 7 (18.4%) | 7 (35.0%) |  | 7 (18.9%) | 7 (33.3%) |  |

The variables were dichotomized using cut-offs selected based on the receiver operating characteristics analysis.

RCS=Royal College of Surgeons Charlson Score; DFI=disease free interval; ICS=immune cell score; PM=pulmonary metastases.

*statistically significant at the level of <0.05

**Supplementary table 2**. Hazard ratios (HR) for 5-year all-cause mortality with 95% confidence intervals in first pulmonary metastases and primary colorectal tumours comparing tertiary lymphoid structures (TLSs) and immune cell score (ICS).

|  | **n** | **HR (95 % CI)** | ***p*** | **HR (95 % CI)** | ***p*** |
| --- | --- | --- | --- | --- | --- |
| **Model 2: TLS density and ICS** | | | | | |
| **Metastases** |  | Crude | | Adjusted* | |
| TLS density (low vs. high) | 65 | 1.13 (0.54-2.35) | 0.742 | 1.20 (0.56-2.54) | 0.642 |
| ICS (low vs. intermediate) | 65 | 0.46 (0.20-1.06) | 0.068 | 0.33 (0.13-0.81) | 0.016 |
| ICS (low vs. high) | 65 | 0.29 (0.09-0.92) | 0.036 | 0.22 (0.06-0.78) | 0.019 |
| **Primary tumours** |  | Crude | | Adjusted** | |
| TLS density (low vs. high) | 54 | 0.45 (0.23-0.88) | 0.019 | 0.43 (0.18-1.02) | 0.055 |
| ICS (low vs. intermediate) | 54 | 0.66 (0.30-1.46) | 0.306 | 0.64 (0.25-1.62) | 0.350 |
| ICS (low vs. high) | 54 | 1.37 (0.55-3.39) | 0.499 | 1.17 (0.35-3.95) | 0.803 |
| **Model 3: TLS max diameter and ICS** | | | | | |
| **Metastases** |  | Crude | | Adjusted* | |
| TLS max diameter (low vs. high) | 65 | 0.67 (0.34-1.33) | 0.252 | 0.74 (0.36-1.52) | 0.406 |
| ICS (low vs. intermediate) | 65 | 0.45 (0.20-1.01) | 0.054 | 0.31 (0.12-0.78) | 0.013 |
| ICS (low vs. high) | 65 | 0.36 (0.13-1.01) | 0.052 | 0.27 (0.08-0.87) | 0.028 |
| **Primary tumours** |  | Crude | | Adjusted** | |
| TLS max diameter (low vs. high) | 54 | 0.31 (0.13-0.70) | 0.005 | 0.31 (0.11-0.84) | 0.021 |
| ICS (low vs. intermediate) | 54 | 0.64 (0.29-1.40) | 0.258 | 0.64 (0.25-1.66) | 0.358 |
| ICS (low vs. high) | 54 | 1.30 (0.53-3.22) | 0.566 | 1.11 (0.32-3.84) | 0.874 |
| **Model 4: TLS hotspot and ICS** | | | | | |
| **Metastases** |  | Crude | | Adjusted* | |
| TLS hotspot (low vs. high) | 65 | 0.76 (0.38-1.52) | 0.436 | 0.93 (0.43-1.99) | 0.843 |
| ICS (low vs. intermediate) | 65 | 0.46 (0.21-1.04) | 0.063 | 0.33 (0.13-0.83) | 0.019 |
| ICS (low vs. high) | 65 | 0.34 (0.12-0.97) | 0.043 | 0.26 (0.08-0.82) | 0.022 |
| **Primary tumours** |  | Crude | | Adjusted** | |
| TLS hotspot (low vs. high) | 54 | 0.49 (0.23-1.03) | 0.061 | 0.50 (0.20-1.25) | 0.139 |
| ICS (low vs. intermediate) | 54 | 0.67 (0.31-1.49) | 0.328 | 0.71 (0.28-1.78) | 0.463 |
| ICS (low vs. high) | 54 | 1.59 (0.63-1.03) | 0.329 | 1.50 (0.43-5.27) | 0.523 |

ICS=immune cell score; TLS=tertiary lymphoid structure.

*adjusted for sex (female/male), age (continuous), RCS (1/≥2), neoadjuvant chemotherapy (no/yes), synchronicity of pulmonary metastases (synchronous/metachronous), number of pulmonary metastases at diagnosis (1/≥2) former liver metastasectomy (no/yes).

**adjusted for sex (female/male), age (continuous), RCS (1/≥2), neoadjuvant chemotherapy (no/yes), CRC stage (I-II/III/IV), CRC grade (1/2/3),.

**
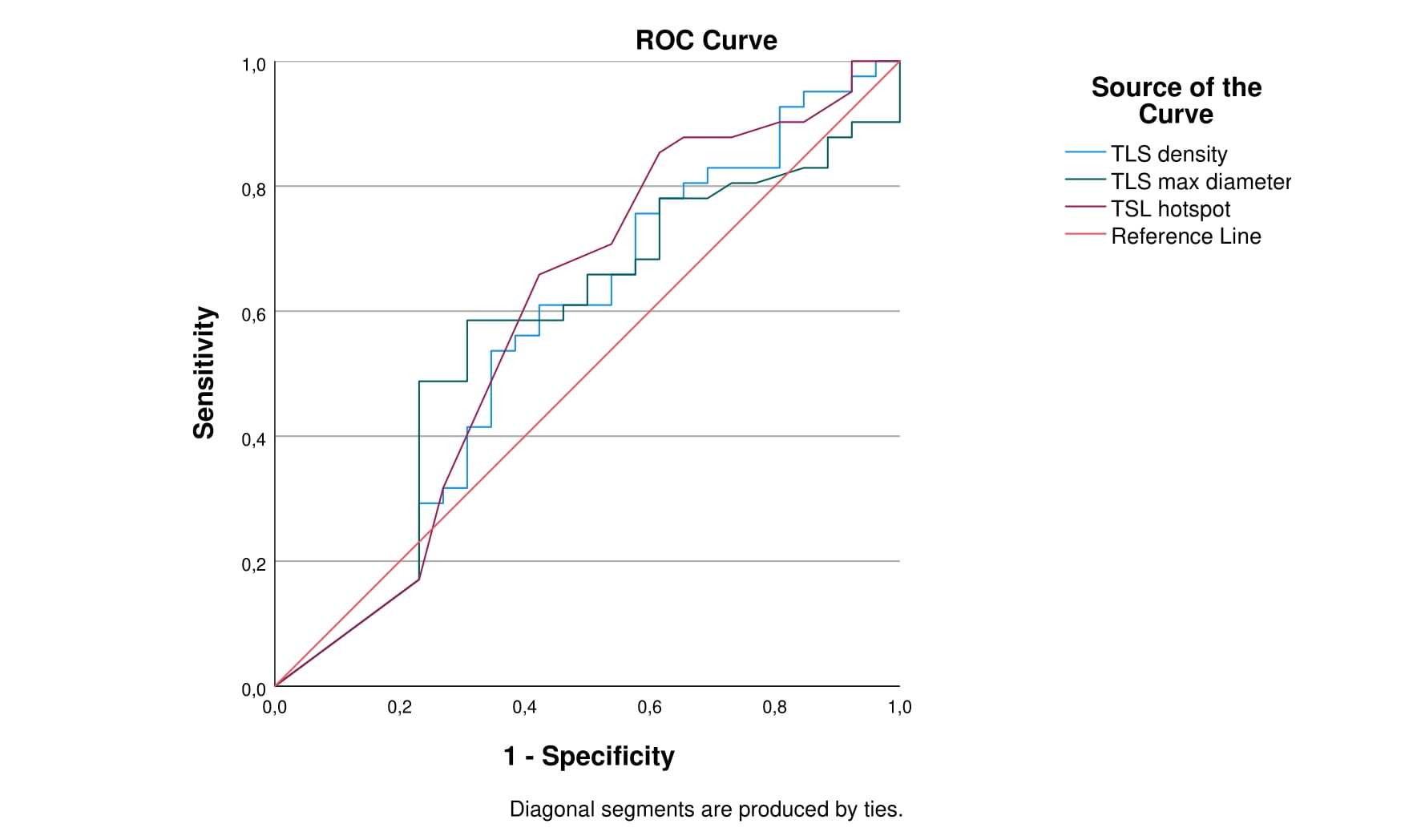
**

**Supplementary figure 1.** Receiver operating characteristics (ROC) curves of TLS measures in the first resected pulmonary metastases of colorectal cancer.

**
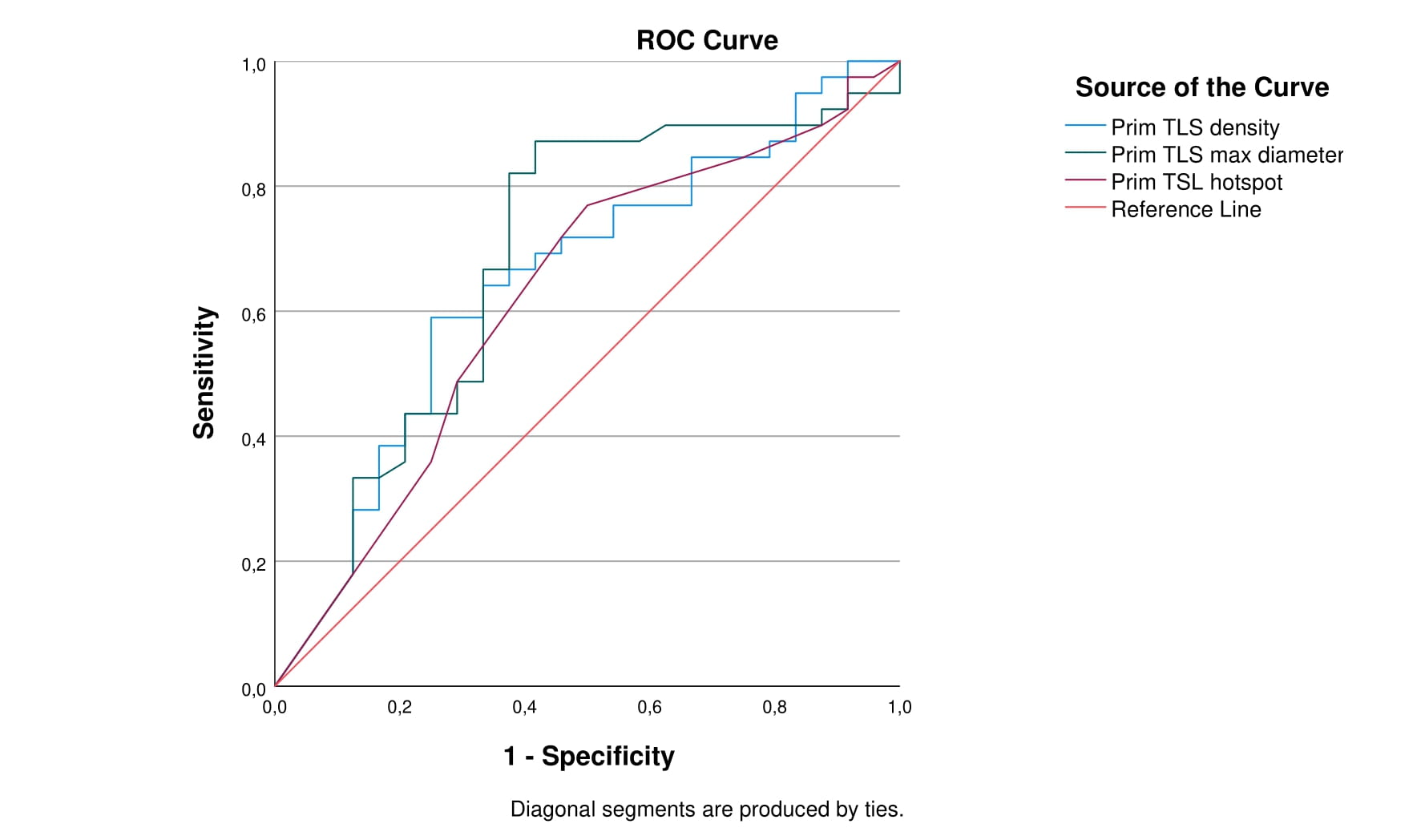
**

**Supplementary figure 2**. Receiver operating characteristics (ROC) curves of TLS measures in the primary tumours.


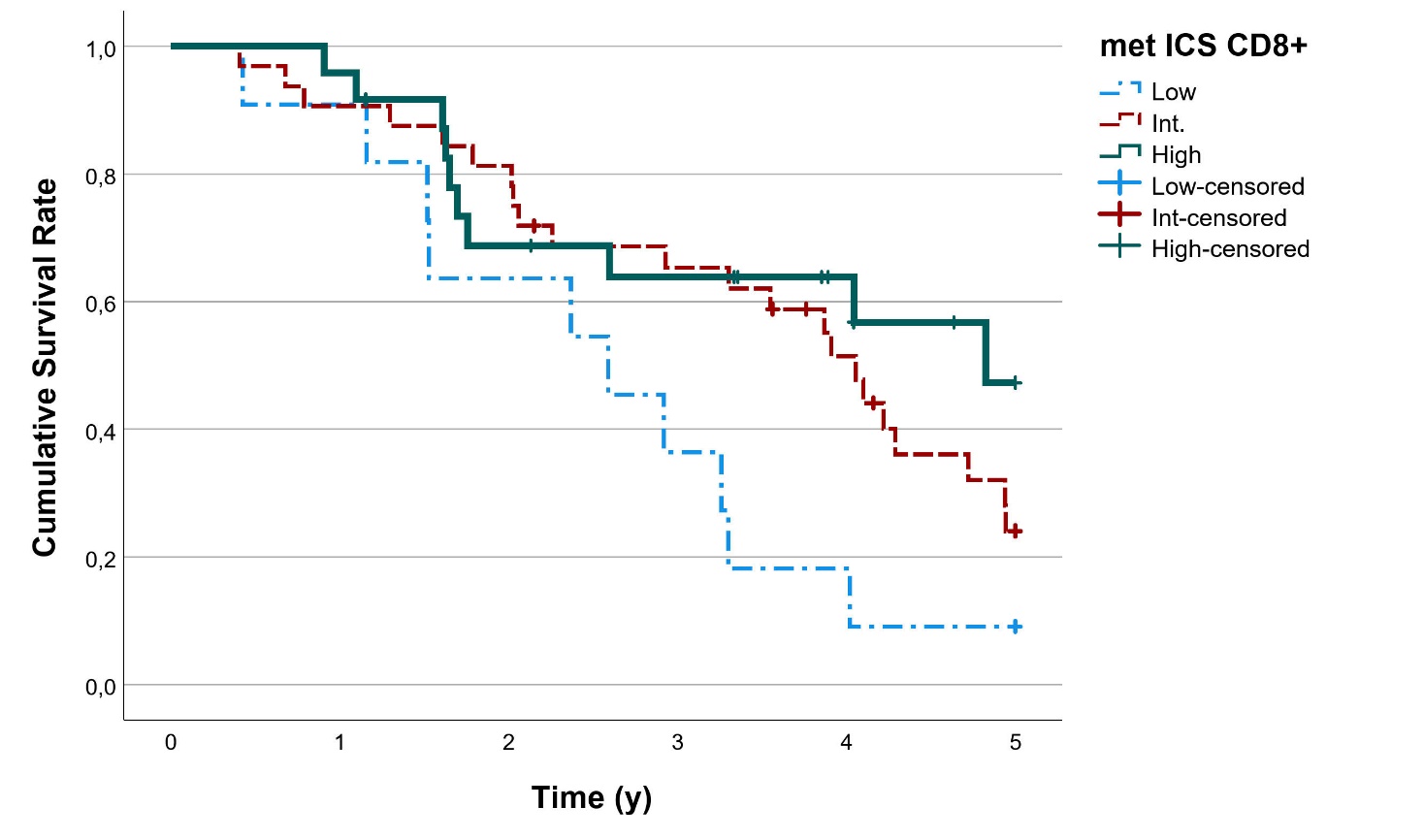


**Supplementary figure 3.** K-M curves of 5-year survival according to CD8+ based immune cell score (ICS) in the pulmonary metastases. Log rank *p=*0.029.
